# Case report: Recreational nitrous oxide abuse triggered peripheral neuropathy possibly through the immune-mediated pathogenesis

**DOI:** 10.3389/fneur.2022.1033327

**Published:** 2022-11-14

**Authors:** Mei-Xue Dong, Qing Wang, Jun-Feng Xu, Ling Hu, Ying Yu, Tao Li

**Affiliations:** ^1^Department of Neurology, Renmin Hospital of Wuhan University, Hubei General Hospital, Wuhan, China; ^2^Department of Neurology, Wuhan No. 9 Hospital, Wuhan, China; ^3^Department of Neurology, Ezhou Central Hospital, Ezhou, China

**Keywords:** nitrous oxide, peripheral neuropathy, ganglioside complexes, Guillain-Barré syndrome, immunoglobin, steroid hormone

## Abstract

Nitrous oxide (N_2_O), commonly known as laughing gas, is widely used in clinical practice and food industry. However, an increasing number of young people have been abusing N_2_O for recreational purpose, resulting in many functional disorders and sometimes irreversible nerve damage. We present the case of a 20-year-old N_2_O abuser who gradually developed peripheral neuropathy after continuously inhaling N_2_O for 2 months. The neurological symptoms of the patient had kept exacerbation for the next 2 months until she came for medical care sitting in a wheelchair. We suggested the patient halting N_2_O intake and supplementing methylcobalamine according to the standardized protocol. Her symptoms had partly recovered during the following 2 weeks but remained unchanged in another 2 weeks. Antibodies against ganglioside complexes were detected and anti-GM1 IgM antibodies were positive in both cerebrospinal fluid and serum. Intravenous immunoglobulin was given as an additional treatment and the patient's symptoms had significantly recovered further. The patient discharged walking by herself. Then she has been continuously followed up in outpatient department for the next 4 months and taking steroid hormone as well as methylcobalamine. Her symptoms gradually disappeared and all the electrophysiological parameters significantly improved. With this case we were able to show that N_2_O-related peripheral neuropathy is not only a metabolic disorder but also an immune-mediated disease. N_2_O intake can trigger a mimic Guillain-Barré syndrome.

## Introduction

Nitrous oxide (N_2_O), commonly known as laughing gas, is a colorless, non-irritating gas with a sweetish smell. It is widely used as an anesthetic in clinical practice and can also be easily obtained in the catering industry for whipping cream preparation and in the motor industry as a fuel booster (1). In the recent years, an increasing number of young people have been abusing N_2_O for recreational purpose. In the UK, N_2_O was the eighth most commonly used substance and its lifetime prevalence was about 38.6% (2). The N_2_O abuse can cause hypotension, lung injury, apnea, and any other accidental injuries with a large dose in a short time. The chronic toxicities of N_2_O are megaloblastic anemia, psychiatric syndromes and neurological injuries, including subacute combined degeneration, myeloneuropathy, myelopathy and peripheral neuropathy (3). To date, the mechanism of N_2_O toxicity has not been clearly elucidated and vitamin B12 deficiency is the most extensively studied mechanism. Vitamin B12 is an important cofactor for methionine synthetase and methylmalonyl coenzyme A mutase and its deficiency can lead to a decrease of methionine, tetrahydrofolate, and S-adenosylmethionine, and an increase of homocysteine, 5-methyltetrahydrofolate, and methylmalonic acid, resulting in nerve demyelination and injury (4). In addition, N_2_O can lead to NMDA antagonism, alteration of cerebral blood flow, and inhibition of the synthesis and release of xanthine and monoamines (5). Here we present the case of a 20-year-old N_2_O abuser mimicking Guillain-Barré syndrome (GBS) with significant immune disturbances.

## Case description

**Figure 1:**
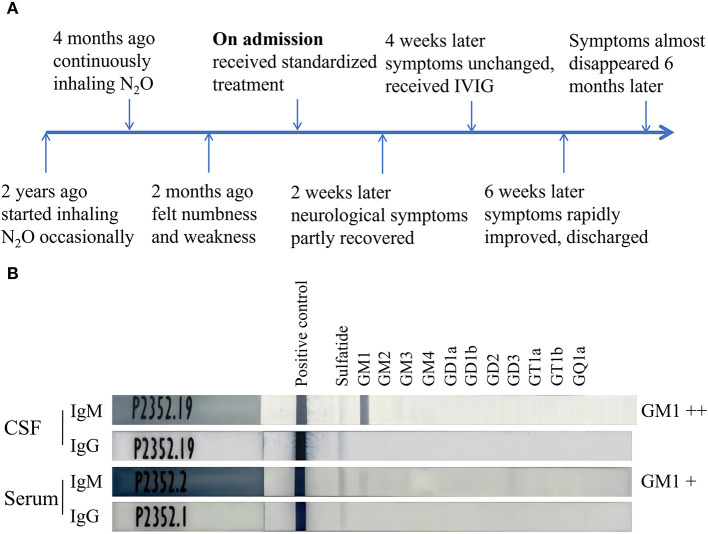
Timeline of the case and ELISA results of antibodies against ganglioside complexes. **(A)** The patient started inhaling N_2_O occasionally 2 years ago, felt numbness and weakness 2 months ago, significantly improved after receiving the treatment of IVIG, and almost recovered 6 months after the admission. **(B)** ELISA tests indicated the anti-GM1 IgM antibodies were positive in both CSF and serum while anti-GM1 IgG antibodies were negative. IVIG, intravenous immunoglobulin; CSF, cerebrospinal fluid.

The patient was a 20-year-old camgirl without any other medical history and occasionally inhaled N_2_O for fun in the last 2 years (Figure 1A). She had kept inhaling N_2_O (about 10 whippets daily) after a failed investment 4 months ago. Two months later she gradually felt numbness and weakness in her lower limbs. She didn't pay attention to it and kept inhaling N_2_O without any treatments. The numbness and weakness kept being heavier and her upper limbs also suffered. The superficial sensation had significantly decreased of both hands and legs below knees. She couldn't feed herself with hands (muscular strength: 3/5) nor stand up (proximal muscular strength: 3/5; distal muscular strength: 0/5), and came for medical care sitting in a wheelchair. Her limbs' muscular tone had decreased and all the tendon reflexes were disappeared while the overall muscle bulks were normal. The deep sensations of vibration, position, and movement stayed normal. Romberg sign and Babinski sign were negative. She had no defecation problems, psychiatric disorders, headache, epilepsy, cognition impairment, or any other neurological symptoms. She also had no history of vaccine injection, enteritis, or influenza in the last year.

Blood tests indicated slightly decreased hemoglobin (129 g/L) and increased mean corpuscular volume (97.8 fL). Plasma homocysteine level was significantly elevated (24.67 μmol/L) while the serum levels of folic acid, vitamin B12, and any other vitamins were normal. Magnetic resonance imaging hadn't found demyelination or any other abnormal focuses in both brain and spinal cord. Electromyography examination showed extensive peripheral nerve damage, involving motor nerve, sensory nerve, and nerve root. Axonal injuries were especially obvious and nerve damages of the lower limbs were significant heavier than that of upper limbs (Table 1).

**Table 1:** Electrophysiological parameters of the case before the treatment with intravenous immunoglobulin.

**Motor nerve**	**Segment**	**Lat (ms)**	**Amp (mV)**	**CV (m/s)**	**Dist (mm)**
Medianus (L)	Wrist-APB	4.00	3.6		55.0
	Elbow-Wrist	8.75	3.6	48.4	230
Medianus (R)	Wrist-APB	4.29	5.1		55.0
	Elbow-Wrist	8.92	4.7	47.5	220
Peroneus (L)	Ankle-EDB	Null	Null		
	Fib.head-Ankle	Null	Null		
Peroneus (R)	Ankle-EDB	Null	Null		
	Fib.head-Ankle	Null	Null		
Tibialis (L)	Ankle-Abd hal	Null	Null		
Tibialis (R)	Ankle-Abd hal	Null	Null		
Ulnaris (L)	Wrist-ADM	3.23	4.4		55.0
	Elbow-Wrist	7.31	4.9	52.7	215
Ulnaris (R)	Wrist-ADM	3.05	5.0		55.0
	Elbow-Wrist	6.79	5.3	56.1	210
**Sensory nerve**	**Segment**	**Peak lat (ms)**	**Amp (uV)**	**CV (m/s)**	**Dist (mm)**
Medianus (L)	Wrist-Dig II	2.35	27.0	48.9	115
Medianus (R)	Wrist-Dig II	2.64	22.3	47.3	125
Radialis (L)	EPL tendon-Wrist	1.65	20.9	54.5	90.0
Radialis (R)	EPL tendon-Wrist	1.50	20.9	66.7	100
Superficial peroneal (L)	Lower leg-Ankle	2.56	3.9	35.2	90.0
Superficial Peroneal (R)	Lower leg-Ankle	2.64	5.4	36.0	95.0
Suralis (L)	Mid.lower leg-Lat.Malleolus	2.19	4.4	38.8	85.0
Suralis (R)	Mid.lower leg-Lat.Malleolus	2.07	3.7	38.6	80.0
Ulnaris (L)	Wrist-Dig V	1.98	24.3	48.0	95.0
Ulnaris (R)	Wrist-Dig V	2.14	26.9	49.1	105
**F wave**	**Segment**	**M-Lat (ms)**	**F-Lat (ms)**	**Amp (uV)**	**F (%)**
Medianus (L)	Wrist-APB	3.5	31.4	59.1	33.3
Medianus (R)	Wrist-APB	4.1	28.5	153	73.3
Tibialis (L)	Ankle-Abd hal	Null	Null		
Tibialis (R)	Ankle-Abd hal	Null	Null		
Ulnaris (L)	Wrist-ADM	2.6	30.2	156	100
Ulnaris (R)	Wrist-ADM	3.3	28.4	224	100

“Null” indicates that no signal can be detected; Lat, latency; ms, millisecond; Amp, amplitude; mV, millivolt; CV, conduction velocity; Dist, distance; L, left; R, right; APB, abductor pollicis brevis; EDB, extensor digitorum brevis; Abd hal, abductor hallucis; Fib.head, fibular head; ADM, abductor digiti minimi; Mid.lower leg, the middle of lower leg; Dig, digitus; EPL, extensor pollicis longus.

N_2_O-related peripheral neuropathy was diagnosed according to the medical history of N_2_O abuse, typical clinical manifestations, and the above auxiliary examinations. Guillain-Barré syndrome (especially acute motor axonal neuropathy, AMAN) should also be considered as differential diagnosis. After all, the patient was suggested to halt N_2_O intake and use methylcobalamine for supplementation according to the standardized protocol. The patient's symptoms had partly recovered during the following 2 weeks. The muscular strength of upper limbs increased to 4/5 while superficial sensation and muscular strength of lower limbs recovered slightly. These syndromes remained unchanged after another 2 weeks.

To obtain optimal treatments further, lumbar puncture was performed and cerebrospinal fluid (CSF) was obtained for tests. The leukocyte count of CSF was 3 cells per μl while the protein level was 0.23 g/L. Antibodies against ganglioside complexes were also detected using a commercial ELISA kit and anti-GM1 IgM antibodies were positive in both serum and CSF while anti-GM1 IgG antibodies were negative (Figure 1B). Intravenous immunoglobulin (0.4g/kg × 5 days) was then given as an additional treatment. The patient's symptoms had significantly improved a week later. Her muscular strength of upper limbs and proximal lower limbs increased to 5/5 when the distal lower limbs increased to 1/5. Her superficial sensation had also partly recovered. The patient discharged from the hospital and could walk independently with a steppage gait and take good care of herself.

Then she has been continuously followed up in outpatient department. Considering the amazing treatment outcome of immunoglobin, she accepted the injection of methylprednisolone (1,000 mg qd × 5 days) and orally took prednisone (60 mg qd × 5 days, 30 mg × 5 days, 15 mg qd × 5 days, 10 mg × 5 days) as follows. After that, she has been continuously taking prednisone 5 mg daily. Four months after the discharge from hospital, her numbness gradually disappeared and the muscular strength of distal lower limbs increased to 4/5. Her gait was almost normal and all the electrophysiological parameters significantly improved. The latencies of motor nerves, sensory nerves, and F waves had extensively decreased while the amplitudes had increased. The incidences of F waves also had significantly increased (Table 2). The patient and her parents were aware of the whole process and pleased with the treatment outcome in spite of the injury induced by lumbar puncture, high price of immunoglobulin, and potential side effects of prednisone.

**Table 2:** Electrophysiological parameters of the case 4 months after the discharge from hospital.

**Motor nerve**	**Segment**	**Lat (ms)**	**Amp (mV)**	**CV (m/s)**	**Dist (mm)**
Medianus (L)	Wrist-APB	3.58	7.1		65.0
	Elbow-Wrist	7.06	6.9	57.5	200
Medianus (R)	Wrist-APB	3.75	8.5		65.0
	Elbow-Wrist	7.54	7.9	60.7	230
Peroneus (L)	Ankle-EDB	Null	Null		
	Fib.head-Ankle	Null	Null		
Peroneus (R)	Ankle-EDB	Null	Null		
	Fib.head-Ankle	Null	Null		
Tibialis (L)	Ankle-Abd hal	5.37	0.082		70.0
Tibialis (R)	Ankle-Abd hal	4.74	0.22		70.0
Ulnaris (L)	Wrist-ADM	2.63	7.2		60.0
	Elbow-Wrist	5.64	7.2	61.5	185
Ulnaris (R)	Wrist-ADM	2.54	10.0		60.0
	Elbow-Wrist	5.31	9.3	61.4	170
**Sensory nerve**	**Segment**	**Peak lat (ms)**	**Amp (uV)**	**CV (m/s)**	**Dist (mm)**
Medianus (L)	Wrist-Dig II	2.25	44.2	55.6	125
Medianus (R)	Wrist-Dig II	2.48	39.1	52.4	130
Radialis (L)	EPL tendon-Wrist	1.51	26.7	59.6	90.0
Radialis (R)	EPL tendon-Wrist	1.35	28.8	66.7	90.0
Superficial Peroneal (L)	Lower leg-Ankle	1.73	14.5	49.1	85.0
Superficial Peroneal (R)	Lower leg-Ankle	1.84	13.1	43.5	80.0
Suralis (L)	Mid.lower leg-Lat.Malleolus	1.87	7.6	42.8	80.0
Suralis (R)	Mid.lower leg-Lat.Malleolus	1.66	11.7	42.2	70.0
Ulnaris (L)	Wrist-Dig V	1.73	45.4	54.9	95.0
Ulnaris (R)	Wrist-Dig V	1.84	43.8	59.8	110
**F wave**	**Segment**	**M-Lat (ms)**	**F-Lat (ms)**	**Amp (uV)**	**F (%)**
Medianus (L)	Wrist-APB	3.4	26.7	267	76.9
Medianus (R)	Wrist-APB	3.6	26.8	463	91.7
Tibialis (L)	Ankle-Abd hal	Null	Null		
Tibialis (R)	Ankle-Abd hal	Null	Null		
Ulnaris (L)	Wrist-ADM	2.5	27.3	189	100
Ulnaris (R)	Wrist-ADM	2.4	25.6	185	100

“Null” indicates that no signal can be detected; Lat, latency; ms, millisecond; Amp, amplitude; mV, millivolt; CV, conduction velocity; Dist, distance; L, left; R, right; APB, abductor pollicis brevis; EDB, extensor digitorum brevis; Abd hal, abductor hallucis; Fib.head, fibular head; ADM, abductor digiti minimi; Mid.lower leg, the middle of lower leg; Dig, digitus; EPL, extensor pollicis longus.

## Discussion

Commonly, recreational N_2_O abuse is recognized to cause metabolic disorders with vitamin B12 deficiency. However, vitamin B12 deficiency is not sufficient to account for N_2_O-induced peripheral neuropathy as these patients showed prominent motor superexcitability changes and less prominent sensory superexcitability changes in nerve excitability test when compared to patients with vitamin B12 deficiency (6). Here, we present the case of a 20-year-old N_2_O abuser suffering peripheral neuropathy mimicking GBS with immune disturbances.

Peripheral neuropathy is the most common N_2_O-related neurological disorder with a morbidity up to 97% (7). These patients primarily exhibited limb numbness or weakness, especially the lower limbs. Decreased muscle strength, superficial sensory disturbances, and decreased tendon reflex were the most common neurological signs according to the published report (7). Increased plasma homocysteine level is more sensitive than plasma vitamin B12 level for clinical diagnosis, as N_2_O mainly consumes vitamin B12 in tissue but not blood (3). Electromyography of these patients indicated mixed axonal and demyelination injury, including motor and sensory nerves simultaneously. Abnormal F wave and H reflex were also found in the majority of N_2_O abuser (8). All in all, the case we presented was a typical N_2_O-related peripheral neuropathy according to these characteristics.

However, without the medical history of N_2_O abuse, these patients were usually misdiagnosed as GBS (9). The absent medical history of vaccine injection, enteritis, or influenza before the onset didn't support the diagnosis of GBS. The course of disease progression of the patient was also significantly longer than the natural history of GBS (2–4 weeks). To our knowledge, GBS is more likely to occur in the middle-aged or aged population but not the youth. The electrophysiological features of N_2_O abuser were dramatically different from those in acute inflammatory demyelinating polyradiculoneuropathy (a GBS variant), but exactly similar to another GBS variant (AMAN). It's difficult to differentiate N_2_O abuse and AMAN according to those electrophysiological findings (10). Meanwhile, the injury severity of lower limbs is usually heavier than the upper limbs in N_2_O abuser while it is similar in patient with GBS (11). The difference in the distal and proximal compound muscle action potential amplitudes of the upper limbs can be a parameter for differential diagnosis between the N_2_O abuser and AMAN patients, as the axonal injury was more severe in AMAN patients than N_2_O abuser, especially upper limbs (10). GBS is an immune-mediated disease while N_2_O-related neurological syndromes are deemed as metabolic disorders. Albuminocytologic dissociation and antibodies against ganglioside complexes in CSF were the specific characteristics of GBS. However, the potential pathogenesis seemed to be more complicated as the motor neuropathy of young N_2_O abuser remained disabled with methylcobalamine supplementation in follow-up research (12).

Here, although the patient was suggested to halt N_2_O intake and supplement methylcobalamine according to the current standardized protocol, her syndromes had only partly recovered without further progress. Lumbar puncture was performed and CSF was tested. Albuminocytologic dissociation was not found in CSF while we were the first to report positive anti-GM1 IgM antibodies in both CSF and serum. Gangliosides are specifically enriched in nervous system plasma membranes while GM1 is mainly expressed at nodes of Ranvier and motor nerve terminals (13). When GM1 or its mimicry, *Campylobacter jejuni* strains, are exposed to the autoimmune system, anti-GM1 antibodies are produced and activate the complement cascade, leading to either reversible conduction failure or axonal degeneration in AMAN patients (14). Anti-GM1 IgM antibodies have also been found in multifocal motor neuropathy, autoimmune limbic encephalitis, Bickerstaff brainstem encephalitis, and any other patients with neuronal or glial damage, resulting in the exposure of GM1 to autoimmune system (15). The disturbed immunological system of the patient with positive anti-GM1 IgM antibodies were probably primarily caused by N_2_O or secondary to the peripheral neuropathy by N_2_O. After all, the favorable prognosis with intravenous immunoglobin and steroid hormone further validated our supposes that immunological system was involved in the N_2_O-related peripheral neuropathy (12). The effect of N_2_O on the neuroimmune system is possibly through vitamin B12 deficiency or any other unclear pathways to be clarified in future (16).

## Conclusion

With this case we were able to show that recreational N_2_O abuse can cause immune-mediated disorder besides metabolic disorder. N_2_O-related peripheral neuropathy is a mimic of GBS with many similar characteristics. We therefore encouraged all clinicians to perform CSF tests of antibodies against ganglioside complexes when encountering N_2_O abusers. The potential immunological pathogenesis should be clarified to find novel treatments for these patients.

## Data availability statement

The raw data supporting the conclusions of this article will be made available by the authors, without undue reservation.

## Ethics statement

The studies involving human participants were reviewed and approved by the Local Ethics Committee of Renmin Hospital of Wuhan University. The patients/participants provided their written informed consent to participate in this study.

## Author contributions

M-XD and TL designed the study and analyzed the patient data. QW, J-FX, LH, and YY collected the original clinical data. M-XD wrote the first draft of the manuscript. All authors commented on previous versions of the manuscript and read and approved the final manuscript.

## Funding

This work was supported by the Open Fund of Hubei Key Laboratory of Renmin Hospital of Wuhan University (2021KFY040).

## Conflict of interest

The authors declare that the research was conducted in the absence of any commercial or financial relationships that could be construed as a potential conflict of interest.

## Publisher's note

All claims expressed in this article are solely those of the authors and do not necessarily represent those of their affiliated organizations, or those of the publisher, the editors and the reviewers. Any product that may be evaluated in this article, or claim that may be made by its manufacturer, is not guaranteed or endorsed by the publisher.
