# Supplementary figures and images for: Case report: Recreational nitrous oxide abuse triggered peripheral neuropathy possibly through the immune-mediated pathogenesis
Source: Front Neurol. 2022 Nov 14;13:1033327. doi: 10.3389/fneur.2022.1033327 (PMC9702802; doi:10.3389/fneur.2022.1033327)

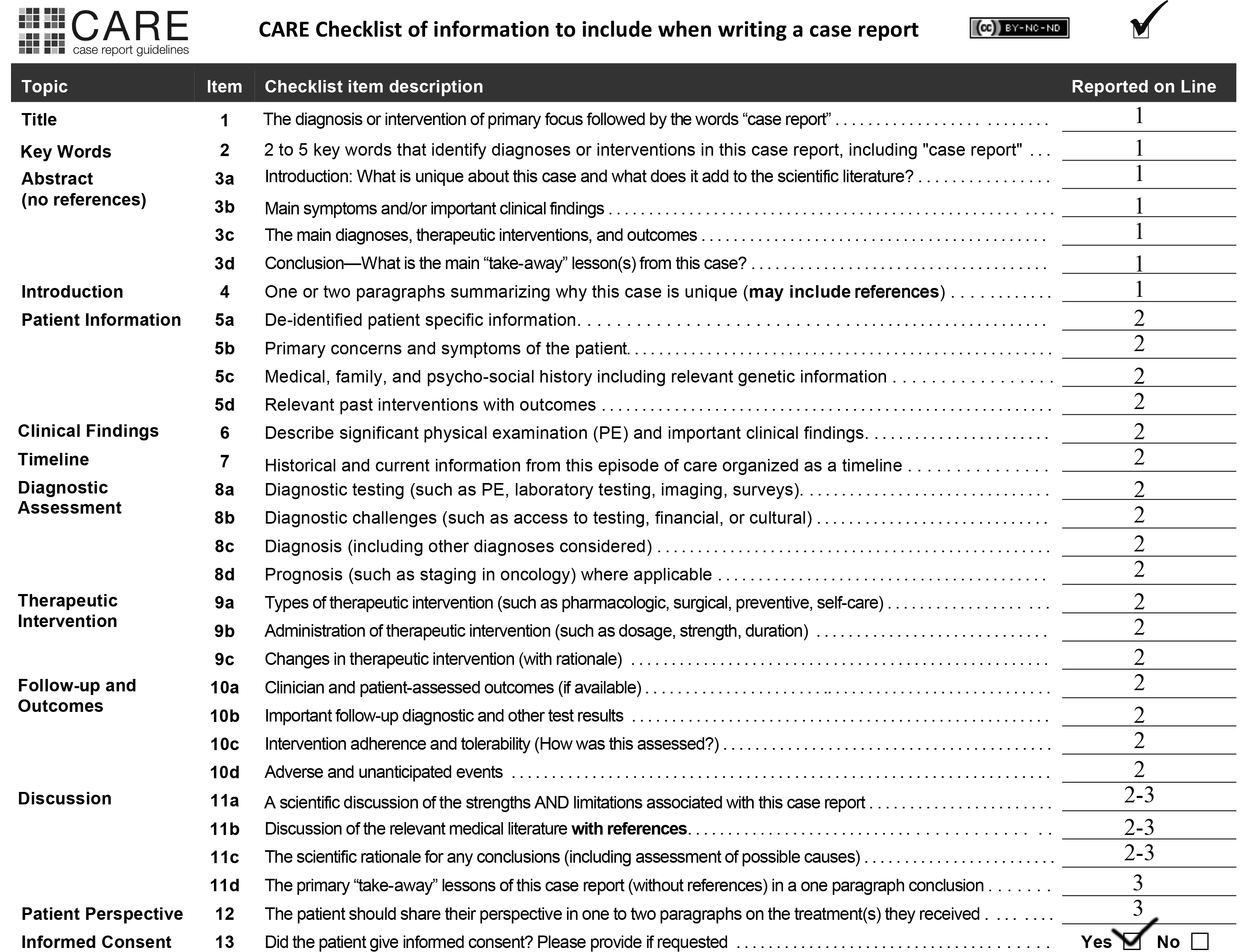

Supplement: Supplementary file 1 [file Image_1.PNG]
